# Discover Your Inner Strength: A Positive Psychological Approach to Bolster Resilience and Address Radicalization

**DOI:** 10.3389/fpsyg.2021.614473

**Published:** 2021-07-14

**Authors:** Mark Dechesne, Jamal Ahajjaj

**Affiliations:** ^1^Dual PhD Center, Faculty of Governance and Global Affairs, Leiden University, Leiden, Netherlands; ^2^Faculty of Social Sciences, Free University of Amsterdam, Amsterdam, Netherlands

**Keywords:** radicalization, resilience, securitization, community, positive psychology

## Abstract

The article reports initial attempts to evaluate a new positive psychological approach to bolster resilience among Muslims in the Netherlands. The approach uses Quranic texts and principles from mental contrasting and implementation intentions (MCII) to encourage Muslims in the Netherlands to reflect in groups on appropriate responses to challenges they are facing. The participants are inspired by Quranic texts and encouraged to write responses to specific challenges in the form of IF-THEN rules and to practice these IF-THEN rules for several weeks. Two studies indicate that this approach increases personal growth initiative. The implications of these findings for the MCII literature and prevention/countering violent extremism are discussed.

## Introduction

Prevention and countering of violent extremism (P/CVE) has become hallmark principles of contemporary counterterrorism and counterradicalization strategy in Europe and (although decreasingly) in the United States. P/CVE goes beyond military effort and law enforcement to also prevent the spreading of violence-propagating ideology and build communal resilience and prevent societal rift. The Countering Violent Extremism Taskforce of the Department of Homeland Security (2020) describes P/CVE as “proactive actions to counter efforts by extremists to recruit, radicalize, and mobilize followers to violence. Fundamentally, CVE actions intend to address the conditions and reduce the factors that most likely contribute to recruitment and radicalization by violent extremists.” By focusing on the stages that precede and follow terrorist acts, CVE implies a broadening of counterterrorism and counterradicalization policy to include a focus on the environments and communities, and on the ideologies, from which radicalization is considered to emanate.

Although hailed as a potential way to address the shortcoming of the strict military and law enforcement approach (Korn, 2016; Selim, 2016), the implementation of PVE/CVE has not been universally positively received. It has been suggested that the social, community approach comes with considerable stigmatization and that ethnic groups have become securitized beyond the terrorist threat that they pose (Choudhury, 2017; Silverman, 2017; Larsen, 2020). Also, with the emphasis on counternarratives, the suggestion has been that religion is a basis for extremism (Choudhury, 2017; De Koning, 2020). As efforts specifically focused on Jihadist inspired forms of radicalizations in P/CVE efforts, resentment increased among a significant share of Muslims that Islamic schools of thought are equated with hostility, violence, and terrorism, and these schools of thought are considered subject of countering and replacement with alternative worldviews (Rashid, 2014; Beller and Kröger, 2020). Not just specific individuals or organizations but communities and religion as a whole have thereby become subjected to security scrutiny (Ragazzi, 2017; Spalek and Weeks, 2017).

In response to this concern, the present contribution makes a plea for a paradigm shift from a threat-based to a positive psychological approach to address radicalization, particularly in the context of Islam. We argue that current PVE/CVE practice derives in part from the assumption of the presence of a threat associated with Islam, which is increasingly difficult to identify as a broader share of the Muslim community becomes implicated in P/CVE programs (e.g., students at schools, mosque attendants, and youth groups). A positive community psychological approach that focusses on the strengths of religion rather than the threat it is alleged to pose may be more effective to realize P/CVE aims without actually seeking to counterradicalization. It is based on the assumption that religion may serve as a framework and source of inspiration for discovering practical ways of dealing with everyday challenges (Costin and Vignoles, 2020). In doing so, it helps to find one’s inner strength and to improve personal effectiveness to deal with these challenges, thereby reducing grounds for societal hostility, and calls for radical change and extremism to bring about that change (Miconi et al., 2020).

For the present special issue, we outline the main ideas behind this positive alternative to P/CVE and report the procedures and preliminary finding of two meeting series in which we introduced the procedures. Based on systematic investigation of self-assessment surveys that were registered during these meetings series, we present preliminary findings showing that bolstering religious commitment may contribute to a sense of personal effectiveness in dealing with challenges faced by Muslims in Netherlands.

### From Threat to Strength

P/CVE encompasses a broad set of initiatives aimed to prevent individuals, groups, and communities at risk of being targeted by radical messaging, from losing connection to society. Initiatives are generally aimed at raising awareness of processes of radicalization, promoting cohesion within communities, and fostering positive contact between communities. These general aims translate in educational programs, knowledge exchange sessions, contact and dialog initiatives,professional training, and community-building projects (Korn, 2016; Spalek and Weeks, 2017; Department of Homeland Security, 2020).

From a psychological angle, these initiatives are aimed to contribute to the establishment of relationship, dialog, and trust (Korn, 2016; Ellis and Abdi, 2017). But many have observed paradoxical results. When applied as broadly as they have been, the projects and initiatives are also directed at individuals, groups, and communities, who are not directly involved in extremism. Consequently, the number of “false positives” concerning “at risk” assessments increases dramatically (Sarma, 2017). Individuals and communities have been approached under the header of CVE with initiatives to establish rapport, dialog, and trust, paradoxically, in the anticipation of a rift that CVE programs are meant to prevent and counter (Ragazzi, 2017; Stanley et al., 2018). For a significant share of “false positives,” this has contributed to a sense of alienation and insecurity, rather than the sense of relationship, dialog, and trust, the programs aimed to establish (Silverman, 2017).

We believe the paradox of the intent to establish rapport, dialog, and trust, essentially to manage a potential threat, has constituted the Achilles-heel of P/CVE initiatives. The root of the problem lies in an unbalanced relational trade-off: Whereas the targets of CVE programs are expected to establish rapport and trust, the initiators of the programs are entitled to act out of distrust. For effective dialog and rapport to emerge, this unbalance needs to be addressed. In order to establish genuine rapport, we need to shift from a threat-based approach, to an approach that focuses on true connection and trust, thereby creating space for dialog (Ellis and Abdi, 2017; Spalek and Weeks, 2017).

Positive psychology has valuable insight to offer in this context. At its very core, positive psychology represents an effort to move away from a focus on the problematic and a move toward the positive (Seligman, 2019). In the context of P/CVE, this essentially translates into a move away from a focus on threat and rather implies a focus on the positive, that is, a focus on the positive features of the groups that are considered to be “at risk.” Although this is a task for the policy makers and practitioners that seek to implement P/CVE interventions, this task can be facilitated by initiatives by the communities that are considered to be “at risk” to show that there is an inner strength, which could be a driving force toward a constructive contribution to society.

This line of thinking represents a paradigm change in the program theory underlying P/CVE intervention. There need not be a change in attitude or shift in identification, or new knowledge (for instance about democracy) to be acquired among the “at risk” population (Feddes et al., 2019). There is also no need to promote contact in order to overcome differences. What is required, rather, is a demonstration that maintaining one’s identity and living by it has positive consequences for one’s participation in and contribution to society.

### Discover Your Inner Strength

From this angle, we started an initiative that we termed “discover your inner strength” with groups of Dutch Muslims. The conceptual background for the program was influenced by existential psychology (Frankl, 1946; Paloutzian, 1981), social learning theory (Bandura, 1977), positive psychology (Duckworth and Seligman, 2017; Seligman, 2019), and health psychology – implementation intentions in particular (Oettingen and Gollwitzer, 2015).

In line with positive psychology, the basic assumption underlying the initiative is that participants in the initiative will be quicker to find positive, constructive solutions to challenges the participants are facing, because the initiative helps to enhance identification with religion and thereby provides inspiration from the “pious predecessors” whose deeds constitute the basis of Quranic narration. The participants are then encouraged to discuss these solutions with fellow attendees and consistent with research on implementation intentions (a cognitive psychological technique not specifically tied to positive psychology), to write the solutions in IF-THEN contingencies whereby the challenge follows the IF and the solution follows the THEN (Gollwitzer and Sheeran, 2006; Kappes and Oettingen, 2014).

This “Salafi” approach (the Salafi approach means practicing faith according to the example of the pious predecessors – the first three generations of Islam) represents a significant departure from common ways in which CVE programs are implemented. For one, a common denominator of these previous programs is the goal of promoting sympathy or support for the “democratic values” or peaceful coalescence (Feddes et al., 2015, 2019). In the current approach, the emphasis is on affirming and bolstering one’s own identity and using it to bring about positive change. Secondly, Salafism itself is often met with suspicion and various analysis point to Salafist ideology as a precursor to support for violent extremism (Wiktorowicz, 2006; Malthaner, 2014; Winter and Muhanna-Matar, 2020). In the present initiative, it is considered a constructive force rather than a driver of isolation, hostility, and extremism.

In doing so, we aim to provide an alternative approach to “counternarratives” that have been employed to redress radicalization. Publicly available empirical evidence on the effectiveness of counterradicalization initiatives is very scarce, but the widespread embrace of the use of counternarratives by policy makers has been described with skepticism (Ferguson, 2016). In one of the very few attempts to systematically study responses to counternarratives, (Bélanger et al., 2021, p. 93) concluded that “countermessages do actually more harm than good” as they showed that presentation of a political countermessage led participants with an obsessive passion for a cause to become more psychological reactant and display greater willingness to engage in violent political behaviors. The Aarhus model to address radicalization (e.g., Ozer and Bertelsen, 2019) relies less on counternarratives and is more based on the idea that strengthening identity development makes one more resilient to radical messages. The assumption is that a clear sense of identity, i.e., a clear sense of who one is, and one’s position vis-à-vis others and society at large, and a clear ability to realize one’s life goals, helps the individual cope with frustrations of life and to contribute constructively rather than destructively to society. But the emphasis in this model is foremost on the creation of personal identity. The potential of social/religious identity to contribute to resilience remains unaddressed, while religion and community are essential resources for validation and affirmation of one’s sense of self and self-integrity, which are crucial for effective goal pursuit and coping with adversity (Greenberg et al., 1986; Baumeister and Leary, 1995).

**Figure 1:**
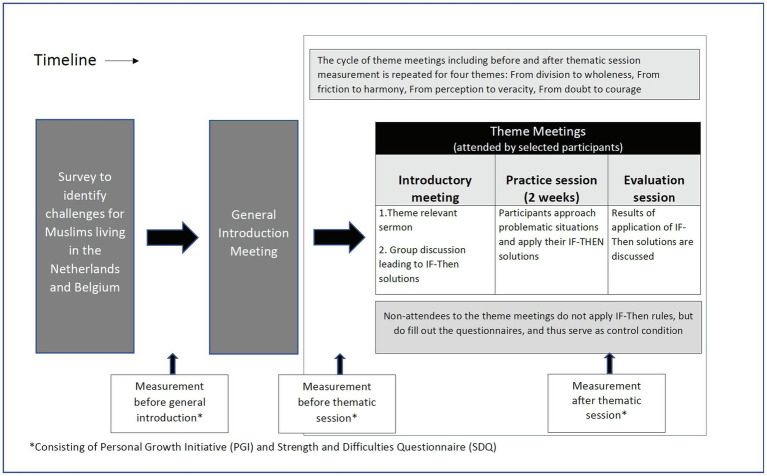
Schematic overview of the design of the intervention.

Thus, the initiative, which is schematically described in Figure 1, was built to identify positive, constructive solutions to basic challenges that the participants were facing using the religious scriptures as a source of inspiration. To obtain a sense of the type of challenges that Muslims in the Netherlands are facing, we put up an announcement of the initiative on a widely attended Web site that provides Islamic content to a Dutch and Belgian audience. The announcement invited volunteers to describe the challenges they were facing as a Muslim in the Netherlands. The researchers then categorized the answers in four categories: challenges related to (1) division among Muslims; (2) friction between Muslims and non-Muslims; (3) negative perceptions by non-Muslims regarding Muslims; and (4) intrapersonal concerns, such as anxiety, self-doubt, and loneliness.

For each of the four categories, positive alternative was identified. In this way, we could organize the initiative around four themes, each of which aiming to establish a positive perspective or goal in response to a challenge. The first concerned the theme “from division to wholeness”; the second “from friction to harmony”; the third “from perception to veracity”; and the fourth “from doubt to courage.”

Each theme was addressed during an introductory meeting, a practice run, and an evaluation session. During the introductory meeting, theme-relevant sermons of the Quran were selected and discussed by the imam involved in the project. The attendees were then seated in groups of 4 to 6 and encouraged to discuss challenges that were related to the meeting theme and to jointly develop effective ways to address these challenges. Attendees were encouraged to use the Quranic sermons as source of inspiration to find effective ways to address the challenge. In line with research on mental contrasting and implementation intentions, attendees were instructed to formulate the outcome of their discussions in the form of “IF … THEN …” rules (Gollwitzer and Sheeran, 2006). The identified challenge was written after the “IF,” and the suggested action to deal with the challenge after the “THEN.” The solutions that were identified were presented to all attendees at the end of the introductory meeting. During the practice run that lasted for 2 weeks, the participants were encouraged to approach the problematic situations and to carry out their IF-THEN plans as they had formulated it. During the evaluation session, the results of the efforts were discussed.

There is considerable evidence that formulating mental contrasts (in this case, the themes “from division to wholeness,” etc.) and implementation intentions (i.e., specific courses of actions to address the identified challenge, for instance associated with division) positively impacts goal commitment and execution of goal-directed behavior (Gollwitzer and Sheeran, 2006). Mental contrasting facilitates accessibility of desired ends, and formulating IF-THEN rules to guide behavior when encountering situations that may undermine the attainment of these desired ends, and thereby promotes the motivation to engage in behavior to achieve this desirable end (Kappes and Oettingen, 2014). Furthermore, according to the theory, the prior consideration of a situated response in terms of an IF-THEN production rule also contributes to the accessibility of a behavioral response, thus facilitating the automatic as opposed controlled execution of the response. Moreover, the simultaneous activation of the goal and situated response is assumed to promote efficacy the with which goals and goal-directed behavior are linked (Kappes and Oettingen, 2014). This simple technique of formulating IF-THEN rules has indeed been shown to enhance effectiveness of health promoting behavior, such as refraining from snacks and exercising, to promote cognitive control, and to enhance self-efficacy (Oettingen and Gollwitzer, 2015).

For the present purpose, we focused our measurement on social and personal control. It was hypothesized that the described procedure would enhance social, personal, and affective control when encountering challenging situations for Muslims in the Netherlands.

## Study 1: First Meeting Series

The first meeting series on this “discover your inner strength” was held between December 2018 and June 2019 at various locations in The Hague, the Netherlands. At a general introductory session held at a mosque, approximately 80 Muslims from different parts of the Netherlands attended. During this general introductory session, attendees were welcomed and explained the general background of the initiative. Participants could indicate their preference for participating in one of the four thematic sessions (as described above), each of which consisting of an introductory meeting, a practice run, and an evaluation session. The meeting series was set up so that one participant would follow one thematic session. However, in actual practice, several who had signed up did not attend, and those who had participated in a previous thematic session showed up in subsequent sessions. In this way, a group of approximately 20 participants were formed who attended the four thematic sessions. Although this contributed to more profound discussion on the methods and experiences of the participants, it did interfere with the envisaged research design.

The initial research design involved repeated survey input from all attendees. It was explained that participants could only attend the general introductory meeting if they had filled out the questionnaire that constituted the basis for the central analyses. The same questionnaire was planned to be filled out by all participants before the general introductory meeting, and for all the four thematic sessions, before the introductory meeting of the thematic session and before the feedback sessions. Thus, all participants in the initiative, regardless of whether they attended a session or not, were initially asked to fill in the same questionnaire nine times.

With this full design, we could have assessed the impact of participating in a specific session (by comparing the scores of those who attended versus those who did not attend) and the impact and durability of this impact of participating in general (relative to not-participating). However, many participants complained about the necessity to repeatedly complete the questionnaire, and because of a change in date for organizational reasons, several invitees were not able to participate in the second thematic session, and hence, it was not feasible to do so.

As a consequence, we focused our attention for the analysis on the first survey administered prior to the general introductory meeting, and the one prior to the introductory thematic meeting, and on the survey prior to the feedback meeting of the first thematic session on “division and wholeness.” Participants filled out the survey that contained questions regarding their ability to grow as a person, i.e., personal growth initiative (Robitschek, 1998). An extensive meta-analysis (Weigold et al., 2020) on the correlates of PGI shows in various clinical, education, and performance-related settings, significant correlations between the construct and reduced reduce distress and enhanced wellbeing in response to stressful and potentially traumatic events. We also measured emotional stability and relatedness to others using an adapted version of the Strength and Difficulties Questionnaire (SDQ) developed by Goodman (2001). We assessed the extent to which participating in the “from division to wholeness” affected these measures.

## Materials and Methods

### Participants

Participation was voluntary, and no monetary reward was given for participating in the study, while attendance to the meeting was free of charge. Although more individuals contributed to the initiative, we received 66 responses to our call to fill out the questionnaires.

This group consisting of 25 male and 41 female Muslims living in the Netherlands (age range: 16–55, with *Mdn* = 26) filled out the questionnaires at least once. Table 1 summarizes the completion behavior of the respondents for the three questionnaires. As can be gleaned from Table 1, only 22 respondents filled the questionnaire three times as requested. The limited number of respondents who completed all three questionnaires is a concern for the main analyses. To obtain a better sense of the specificity of the finding to the limited set of participants, we will consider not only the participants who filled out the questionnaires during all three time periods but also those who completed the questionnaire at measurement 3, after having completed one prior questionnaire.

**Table 1:** Number of respondents who completed the questionnaire.

Measurement	*N*	Percentage
Before general introduction session only	11	16.7%
Before thematic session only	13	19.7%
After thematic session only	6	9.1%
Before general introduction session and before thematic session only	7	10.6%
Before thematic session and after thematic session only	5	7.6%
Before general introduction session and after thematic session only	3	4.5%
Before general introduction session, and before thematic session and after thematic session	21	31.8%
Total	66	100%

### Procedures

The general introduction session was held at the main prayer room of a large Mosque in The Hague. Approximately 80 participants attended the meeting. They were welcomed by the two organizers. The organizers explained that the initiative was about finding positive solutions to challenges that Muslims in the Netherlands are facing in their everyday life. The procedure and underlying ideas were explained in considerable detail. One of the organizers talked about personal experiences and how faith served as a source of strength and inspiration. The other organizer explained in general terms how the four thematic clusters of challenges faced by Muslims in the Netherlands were identified, explained the conceptual background and procedures of the mental contrasting and implementation intention method, and explained the rationale behind the questionnaire as a tool to monitor the impact of the procedure. Participants were then assigned to one of the four thematic sessions and informed about the content and procedure of the thematic sessions. It was stressed that participation was on voluntary basis and participants were free to withdraw from the study at any point in time.

The thematic session “from division to wholeness” was the first in the series and held at a community center of an Islamic organization in The Hague. Attendees were welcomed by the organizers and seated by gender at tables of 4 to 6. After a general welcome, the attendees were explained that their input on challenges related to division within the Muslim community had led the imam to select the surah *Ale-Imran*, verse number 103, as a fitting text to reflect on the theme. The surah states:

And hold fast, all of you together, to the Rope of Allah (i.e., this Qur’an), and be not divided among yourselves, and remember Allah’s Favor on you, for you were enemies one to another but He joined your hearts together, so that, by His Grace, you became brethren (in Islamic Faith), and you were on the brink of a pit of Fire, and He saved you from it. Thus Allah makes His Ayat (proofs, evidences, verses, lessons, signs, revelations, etc.,) clear to you, that you may be guided.

After a reflection by the imam on the verse, participants were encouraged to discuss how this verse may provide inspiration for addressing day-to-day challenges and to provide a summary of the discussion in IF … THEN … format. Specifically, they were asked to write the challenge they were facing after the “IF” and their solution after the “THEN”; hence, “IF I encounter this challenge THEN I should do…” Each group formulated several of these IF THEN statements, mostly more than six statements. Examples of these statements include “IF I have a disagreement with my parent regarding religion THEN I treat my parents with respect and discuss my viewpoint in an open manner” and “IF I know of fellow Muslims who refuse to go to my Mosque THEN I will search for a respected mediator to explore the possibility of reconciliation.” Each table then presented the results during a general discussion. Attendees were encouraged to put in practice the IF THEN rule they had formulated during the 2 weeks after the meeting.

After the 2 weeks of practice, participants (both those who had attended the thematic session and those who had not) were sent an email encouraging them to complete the questionnaire once more.

### Questionnaire

The introduction of the questionnaire stressed that all data would be treated confidentially. It was noted that attendees were asked to write their names on the forms but this was only done to be able to link the scores of the multiple measurements. Participants were instructed to indicate their endorsement with statements using a 7-point scale whereby 1 indicated completely disagree and 7 indicated completely agree. The statements were derived from the existing questionnaires. We asked participants to indicate their endorsement of statements pertaining to their PGI (Robitschek, 1998). This scale includes items, such as “I know how to make a realistic plan to change myself,” “I actively work to improve myself,” and “I look for opportunities to grow as a person.” We also asked participants to indicate their (dis)agreement with an adapted version of the SDQ (Goodman, 2001). Although originally meant for diagnosis of psychiatric disorder in children, we thought the statements were suitable to measure emotional wellbeing and sociability, regardless of age of the participant. For instance, items include “I feel sometimes afraid, and I am easily scared,” “I am easily distracted,” and “I am aware of the feelings of others.”

## Results

### Factor Analysis

Principle component factor analyses were conducted on the PGI and SDQ for a consistent set of items. For the PGI, we found that most items loaded on a single factor. Items with a factor loading < 0.40 were deleted from the composite score that was used for further analyses. The SDQ revealed a two-factor structure with a first factor clustering items together that all appear to be related to emotional stability and a second factor clustering items together that appear to pertain to sociability. Hence, we used personal growth initiative, emotional stability, and sociability as the three scales to investigate the impact of participation in the thematic session.

### Personal Growth Initiative

**Figure 2:**
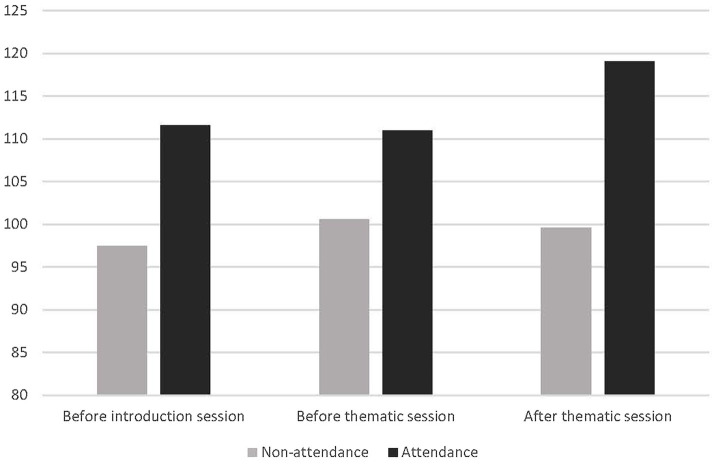
Mean personal growth initiative (PGI) scores in Study 1 as a function of timing of measurement attendance (versus non-attendance) to a thematic session and practice afterward. Higher cores indicate greater personal growth initiative. Scores could be from 22 (lowest) to 154 (highest).

The PGI composite scores were subjected to a 2 (Attendance: Attendance versus No attendance at the thematic session) between-subjects × 3 [Measurement: (1) Before general introduction vs (2) Before thematic session vs (3) After thematic session and practice] within-subject ANOVA. This analysis yielded a significant main effect for Measurement, *F*(2, 14) = 6.78, *p* < 0.01, with a hint of a qualification by Attendance × Measurement interaction, *F*(2, 14) = 2.55, *p* < 0.12. Figure 2 provides the relevant means.

Analysis of participants who completed the questionnaires before the general introduction meeting and after the thematic session and practice (i.e., measurements 1 and measurement 3) showed a similar effect with a main effect for measurement *F*(1, 19) = 8.66, *p* < 0.01, but now with a significant interaction, *F*(1, 19) = 7.14, *p* < 0.02. An analysis of participants who only completed the questionnaires before and after the thematic session but not before the general introduction session (i.e., measurement 2 and measurement 3) also revealed a similar pattern, now without a significant main effect, *F*(1, 20) = 0.96, *p* < 0.34, but with a significant interaction, *F*(1, 20) = 7.39, *p* < 0.02.

If there was an effect of the intervention, we would expect this to occur for those participants who attended (vs. those who did not attend) the thematic session and had engaged in the practice session. For participants in the thematic session, we expected greater PGI after the participants had attended the session and after practice.

Follow-up analyses confirmed this. Participants who attended the thematic session and practiced showed higher PGI after the session and practice than before. Comparing the scores from the general introduction meeting to the post thematic session and practice measure (i.e., measurement 1 vs. 3), the difference is *F*(1, 11) = 19.92, *p* < 0.002, with *M* = 109.91 and *SD* = 14.28 at measurement 1 vs. *M* = 116.83 and *SD* = 13.27 at measurement 3. The corresponding *F*-value for the measurement 2 vs. measurement 3 comparison is *F*(1, 10) = 13.79, *p* < 0.005, with *M* = 110.36 and *SD* = 13.44 at measurement 2. Participants who did not attend the thematic session showed no increase in PGI at measurement 3 relative to measurement 1, *F*(1, 8) = 0.029, *p* > 0.86, nor relative to measurement 2, *F*(1, 10) = 1.00, *p* > 0.34, with respective statistics, *M* = 103.67 and *SD* = 19.64 at measurement 3 vs. *M* = 103.33 and *SD* = 23.33 at measurement 1, and *M* = 108.10 and *SD* = 17.58 at measurement 2. Participants who attended showed higher PGI relative to those who did not attend, but only after the thematic session had taken place, *F*(1, 30) = 7.61, *p* < 0.01. Before the session, there were no differences between attendance versus no attendance groups, *F*(1, 40) = 0.052, *p* > 0.81 at measurement 1, and *F*(1, 41) = 0.224, *p* > 0.63 at measurement 2, suggesting the observed differences cannot be attributed to pre-selection.

### Emotional Wellbeing

**Figure 3:**
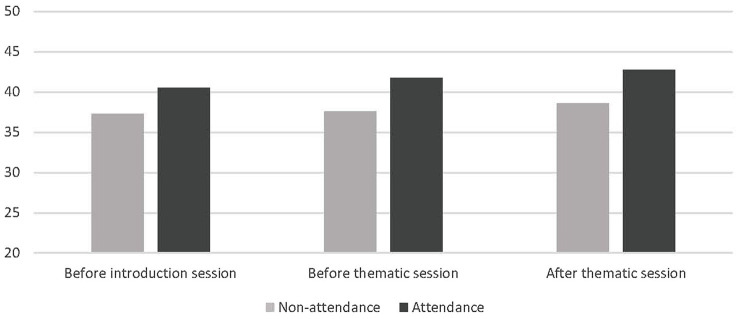
Mean emotional wellbeing scores in Study 1 as a function of timing of measurement attendance (versus non-attendance) to a thematic session and practice afterward. Higher cores indicate greater emotional wellbeing. Scores could be from 8 (lowest) to 56 (highest).

We also performed a 2 (Attendance) between-subjects × 3 (Measurement) within-subject ANOVA on the emotional wellbeing subscale of the SDQ. This analysis yielded a marginally significant main effect of Attendance, *F*(2, 15) = 3.19, *p* < 0.08, but no sign of an interaction of Attendance and Measurement, *F*(2, 15) = 0.437, *p* > 0.65. Figure 3 depicts the relevant means. The main effect of Attendance appears to indicate that irrespective of the time of measurement, participants who did not attend the thematic session and engaged in practice reported less wellbeing than those who did. But given that this differences were already observed twice before the thematic session and practice, this difference is likely to have been caused by a selection mechanism that appears irrelevant for the purposes of the study.

### Sociability

Similarly, to the emotional wellbeing scale, there were also no significant main effects nor interactions for a 2 (Attendance) × 3 (Measurement) ANOVA performed on sociability. We found no indication of a main effect, *F*(2, 18) = 0.41, *p* > 0.95, nor of an interaction, *F*(2, 18) = 0.73, *p* > 0.92. Because of the very limited yield of this analysis, we will not discuss it further.

## Discussion

The results of the first study to assess the impact of the “discover your inner strength’ initiative on participants’ personal growth initiative, emotional wellbeing, and sociability provided some encouraging results. Although we did not find effects of the procedures on emotional wellbeing and sociability, we did register tentative evidence that addressing challenges faced by Muslims in the Netherlands by using Quranic texts as a source of inspiration to define implementation intentions to achieve a desired stated, as our approach aimed to achieve, contributes to greater positive growth initiative among participants who had participated in the critical meeting and practices that followed the meeting relative to participants who had not attended.

Although this effect was observed using a fairly limited number of study participants, we nonetheless were encouraged by it. A thorough discussion on the experiences of the participants held during a session after the practice and final measurement corroborated the statistical findings. Participants indicated that they had a sense of greater control while dealing with their challenges, that they had a clearer sense of an appropriate course of action when confronted with a challenges, and that, as a result of the thematic session, they were better able to focus while being confronted with the challenge.

A side benefit of the procedure, which perhaps may ultimately prove more important than the effect of attendance on personal growth initiative, was that throughout the initiative, from reading the Quranic text, to jointly discussing challenges and formulating actions to deal with the challenge, to being encouraged to engage with the challenge, to reflecting on the results, the used procedures were found to serve as a shared frame of reference, enabling dialog about very sensitive and personal matters. This is currently an underreported benefit of the ever expanding literature on mental contrasting and implementation intentions. Often, sharing thoughts and experiences can be a critical element in effective goal pursuit, whether it be social, health-related, or otherwise, and formulating challenges in terms of IF THEN rules may be a powerful elicitation tool to address difficult personal and social matters.

Notwithstanding these positive effects, we should stress that the reported results are only very preliminary. We reported on a fairly small sample. Also, we only reported the findings regarding one theme, i.e., from division to wholeness, and not regarding the other three. Furthermore, although we did informally observe increasing positivity regarding the initiative and the use of the mental contrasting and implementation intention methods, this may also be caused by an increased self-selection, with those with affinity with the procedures staying within the initiative and those without affinity dropping out. Regarding these matters, conducting more studies with different populations seems to be the only remedy.

## Study 2: Second Meeting Series

A second study was conducted as part of a “discover your inner strength” series held at a Mosque in Rotterdam. This series was more concise than the one reported in Study 1. There were four sessions, with each of the sessions addressing a theme (i.e., “from division to wholeness,” “from friction to harmony,” “from negative perception to veracity,” and “from doubt to courage”). The first session started with a general introduction of the meeting series, and the other three sessions included a 15-min opportunity at the beginning of the session for the participants to discuss their experiences while applying the IF THEN rules to the challenges they were facing. A week prior to each of the meetings, participants filled out a questionnaire with questions related to their personal growth initiative, their emotional stability, and their sociability. Our primary interest was in linear incremental trends for these dependent variables as the meetings progressed.

As a precautionary note for the data analysis, although our initial plan was to track the evolution of these scores during the meeting series, not all participants responded for all sessions to the call to fill out the questionnaires. Hence, the envisioned repeated-measures analyses could only be applied to a very limited set of participants, and we therefore based our analyses primarily on group-level comparison between sessions than individual-level comparison.

## Materials and Methods

### Participants

Participants were 17 males and 31 females (age range: 15–54, *Mdn* = 29) who had signed up for the initiative as announced *via* the communication channels available to the Mosque where the sessions took place. Participants paid an attendance fee of 30 Euros for the entire meeting series.

### Procedure

The procedures and materials were generally the same as used in Study 1. This time, there were four sessions, each addressing a theme (“from division to wholeness,” “from friction to harmony,” “from negative perception to veracity,” and “from doubt to courage”). The sessions were scheduled approximately 4 weeks apart.

The first session started with a general introduction on the meeting series and the rationale behind it, including a brief description of the mental contrasting and implementation intentions method. The final session included a brief report on the research findings and a general group discussion concerning the merits of the meeting series. Otherwise, the four sessions had a similar protocol, whereby the imam introduced the specific theme of the session and narrated a relevant surah, after which participants were divided in groups of 4 to 6, encouraged to discuss challenges and ways to deal with the challenges using the surah, and to write IF THEN contingencies and report these to the entire group at the end of the session. There was a 15-min comfort break planned in each session occurring at variable times during the meetings.

For the first session on the theme “from division to wholeness,” the imam discussed a Hadith of the Prophet Muhammad saying:

Believers are like one body in their mutual love and mercy. When one part of a body is in bad health, the rest of the entire body joins it in restlessness and lack of sleep and is busy with its treatment. Likewise, Muslims should run to helping each other.

For the second thematic session on “from friction to harmony,” the imam discussed surah Ale-Imran, verse number 159:

And by the Mercy of Allah, you dealt with them gently. And had you been severe and harsh-hearted, they would have broken away from about you; so pass over (their faults), and ask (Allah’s) Forgiveness for them; and consult them in the affairs. Then when you have taken a decision, put your trust in Allah, certainly, Allah loves those who put their trust (in Him).

For the third session on “from perception to veracity,” the imam discussed surah AL-HUJURAAT, verse number 6:

O you who believe! If a rebellious evil person comes to you with a news, verify it, lest you harm people in ignorance, and afterward you become regretful to what you have done.

For the fourth session concerning the theme “from doubt to courage,” the imam discussed a Hadith of the Prophet Muhammad saying:

A strong believer is better and is more lovable to Allah than a weak believer, and there is good in everyone (but) cherish that which gives you benefit (in the Hereafter) and seek help from Allah and do not lose heart, and if anything (in the form of trouble) comes to you, do not say: If I had not done that, it would not have happened so and so, but say: Allah did that what He had ordained to do and your “if” opens the (gate) for the Satan.

A week prior to each of the sessions, participants were approached *via* email, reminded about the upcoming session, and encouraged to fill out a digital questionnaire.

### Materials

The digital questionnaire was virtually identical to the one used in Study 1. It asked about the names and gender of the participants and assured that at no point a connection would be made between the participant’s name and the questionnaire scores. The questionnaires were the same as Study 1, i.e., the PGI questionnaire (PGI), and a 7-point Likert version of the SDQ that we analyzed in terms of emotional wellbeing and sociability dimension.

## Results

### General Data Analytic Considerations

The main aim of the data analysis was to statistically establish incremental trends in PGI and emotional wellbeing and sociability, as a result of participating in the meeting series. We were expecting that participants would attend all meetings and fill out all questionnaires. However, this turned out to be too optimistic as some individuals did not attend all sessions, and not all individuals who attended the sessions had filled out the questionnaires. Instead of focusing exclusively on the limited set of individuals who did attend all sessions and who filled out all questionnaires, we decided to track the development of the dependent variables of interest on a group level by comparing mean scores between sessions. Unfortunately, only a very limited group of participants filled out the final questionnaire that was included prior to the last thematic session, and as a result we could only compare the scores for the pre-sessions baseline, the scores measured prior to the second session, and the scores measured prior to the third session.

### Personal Growth Initiative

**Figure 4:**
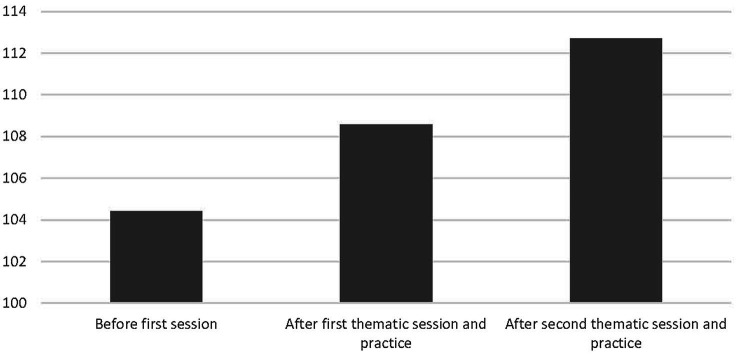
Mean PGI in Study 2 as a function of repeated attendance of a thematic session and practice afterward. Higher cores indicate greater personal growth initiative. Scores could be from 22 (lowest) to 154 (highest).

There were seven participants who had completed the PGI questionnaires for all 3 measurement periods. We first conducted a 3[Measurement: (1) Before introduction session vs (2) After the “division to wholeness” thematic session and practice vs (3) After the “friction to harmony” thematic session] repeated-measures ANOVA on the PGI scores of these seven participants, yielding an overall effect of *F*(2, 5) = 1.96, *p* < 0.23, and more relevant for present purposes, a marginally significant linear trend effect of *F*(1, 6) = 3.67, *p* < 0.11. Relevant means are displayed in Figure 4. From Figure 4, it can be inferred that attendance linearly increased self-reported PGI. We also conducted a 3(Measurement) between-subjects ANOVA with polynomial contrasts on PGI to investigate whether the observed within-subject effect among the seven participants could also be observed on a group level (now with *n* =31 at measurement 1, *n* = 28 at measurement 2, and *n* = 16 at measurement 3). We indeed observed a similar linear trend, *F*(1, 72) = 3.26, *p* < 0.08, with an overall effect of *F*(2, 72) = 1.67, *p* < 0.20. This linear between-groups effect reflects that, in correspondence with the pattern depicted in Figure 4, PGI increased as the meeting series progressed, with *M* = 106.94 and *SD* = 17.59 at measurement 1, *M* = 108.82 and *SD* = 15.13 at measurement 2, and *M* = 115.81 and *SD* = 13.93 at measurement 3. This finding corroborated the results reported in Study 1.

### Emotional Wellbeing

**Figure 5:**
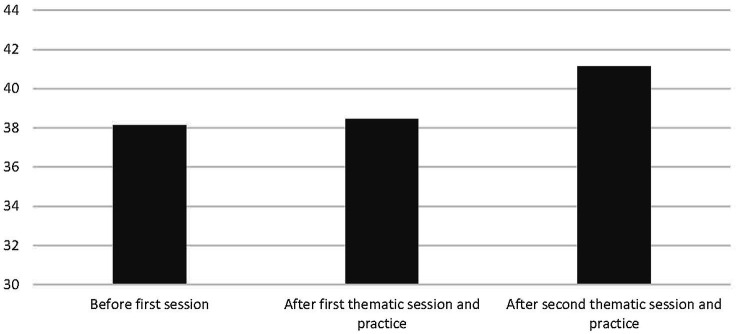
Mean emotional wellbeing scores in Study 1 as a function of repeated attendance of a thematic session and practice afterward. Higher cores indicate greater emotional wellbeing. Scores could be from 8 (lowest) to 56 (highest).

We conducted similar analyses for the emotional wellbeing component of the SDQ. For the participants who completed all three measurements, a 3(Measurement) within-subjects ANOVA using polynomial contrasts showed an indication of a linear effect, *F*(1, 6) = 4.11, *p* < 0.09, with an overall effect of *F*(2, 5) = 2.61, *p* < 0.17. As can be seen in Figure 5, this effect indicates a tendency of the participants to report higher wellbeing as the meeting series progressed. However, using a between-groups ANOVA with a substantially higher number of respondents, this effect was no longer observed, with an overall *F*-value of *F*(2, 72) = 0.25, *p* > 0.78 and a linear effect of *F*(1, 72) = 0.25, *p* > 0.61. Given the inconsistency between the within- and between-subjects comparison, we are cautious to draw any conclusions regarding these emotional wellbeing findings.

### Sociability

We also conducted a 3(Measurement) within-subjects ANOVA on the Sociability dimension of the SDQ, but failed to detect any significant differences, neither for the overall effect, *F*(2, 5) = 0.26, *p* > 0.78, nor for the linear trend, *F*(1, 6) = 0, ns. The between-groups comparison also failed to show indication of a difference, *F*(2, 73) = 0.21, *p* > 0.81, nor of a linear trend, *F*(1, 73) = 0.04, *p* > 0.83.

## Discussion

Study 2 provided us with the opportunity to evaluate the finding from Study 1 that participating in the initiative and engaging in mental contrasting and implementation intention increases one’s perceived personal growth initiative. The findings of Study 2 tentatively confirmed this. During the meeting series of Study 2, we observed rising levels of PGI as the meeting series progressed. We are aware that these findings are tentative. First, the observed incremental trend as a result of prolonged meeting participation was only marginally significant. Secondly, the comparison was based on a small sample within-subject comparison and a less than ideal between-groups design rather than a within-participants repeated measurement, although some of the participants in the sample did participate in all the sessions, whereas others only participated in one or two sessions.

However, we do find the presently reported findings noteworthy. Most importantly, the findings show a virtually identical pattern to the findings reported under Study 1, with statistics indicating that participating the meeting series contributed to a greater sense of personal growth initiative, but not to greater emotional wellbeing nor sociability. Of interest, the findings of Study 1 pertained solely to the effect of participating in a theme meeting related to “from division to wholeness,” whereas the findings of Study 2 pertained to the effects of participating in two thematically different theme meetings, i.e., one of “from division to wholeness” and another on “from friction to harmony.” We also find the findings of Study 2 noteworthy because in an evaluation that was part of the final session of the meeting, many of the participants pointed to exactly improved PGI as the main benefit of the sessions. One mentioned that he had a better sense of what to do when facing important challenges. Another mentioned she felt better prepared when confronted with a challenge. And yet another felt more in charge of the situation.

## General Discussion

This article presented findings from two initial attempts to develop a positive psychological approach to empower Muslims in the Netherland to address social challenges they are facing. The research comprising two studies showed that the combination of Quranic surahs and the psychological procedures of mental contrasting and implementations intentions contributes to a greater sense of PGI (and not to greater emotional wellbeing and sociability) that Muslims in the Netherlands are experiencing.

As this article is part of a special issue on radicalization, we emphasize that the presented initiative is significant in the context of P/CVE programs (Korn, 2016; Selim, 2016). Whereas many P/CVE programs are focused on providing alternative standards (Braddock and Horgan, 2016), here we show that affirmation of religious values may have positive consequences for adjustment and addressing challenges. The research was not conducted nor presented to the participants as an attempt to counter or prevent radicalization. Nonetheless, we do believe the procedures and materials are of value in the context of P/CVE as they reveal a positive way to address challenges, such as division, friction, negative perceptions, and doubt, as well as lack of personal efficacy, challenges that are often considered to create an openness for radical messages (see, e.g., Kruglanski et al., 2019 for review).

We acknowledge the limitations of the present findings. First, the studies involved fairly small samples, and as participation was on voluntary basis, there may have been a selection bias in terms of the type of participants who signed up for the initiative, as well as the type of participants who decided to fill out the questionnaires. As a result, there is still much to learn regarding the reception of the initiative among groups that may initially be less open. Secondly, in both studies, the limited number of participants created less than optimal conditions for statistical analyses.

We also acknowledge that given the nature of the design, we are unable to draw firm conclusions regarding the efficacy of the current procedure relative to other procedures. Our claim has been that the presently advanced positive psychological community-based approach has merits relative to other interventions. Yet, in the absence of a parallel intervention that lacks the characteristics of our presently described intervention, we cannot empirically substantiate this claim regarding merit. Further, in the absence of another intervention, we cannot completely rule out that the presented results emanate from demand characteristics could be responsible for the observed increases in PGI with participants spending multiple sessions focused on meeting challenges becoming more likely to believe that their personal growth should be higher. There may also be the element of self-perception at work, as people who witness themselves repeatedly engaged in these exercises could come to believe that growth is occurring. However, although feasible, we do believe these issues of demand characteristics and self-perception are less likely to apply because it is not clear why these issues would apply specifically to our PGI measure, and not to our sociability nor our emotional wellbeing measure. In general, we recognize that the presently reported findings are therefore only an initial step in assessing the merits of the initiative for promoting personal growth initiative, and more broadly, for building resilience among Muslims to face the challenges they are facing in Western countries, such as the Netherlands.

Notwithstanding these shortcomings, we do think the initiative deserves broader consideration. One reason is the consistency in results, showing that participating in the series contributed to personal growth initiative. But there were other indications that the initiative made a positive contribution to the lives of the participants. Although privacy considerations brought us to refrain from formally recording the conversations about the experiences of the participants, these conversations did reveal that the method of mental contrasting and implementation intention had been instrumental in effectively addressing a number of the challenges that the participants had brought up. As already alluded to, multiple participants reported they had developed a better sense of the underlying reasons of the conflict they had experienced, they felt better prepared when facing the challenges, and they also felt supported that they could share and discuss their experiences.

In this latter sense, we believe we have identified a previously underreported merit of mental contrasting and implementation intention method, particularly when it is applied to sensitive issues (Gollwitzer and Sheeran, 2006; Oettingen and Gollwitzer, 2015). The program, especially when combined with an identify affirming narrative, creates a frame that enables a (in our experience) very constructive dialog about difficult matters (such as division, friction, negative perception, and doubt). The participants generally felt support simply by discussing their experiences with likeminded others. The dialog contributed to a shared reality and affirmed identity, while promoting efficacy in dealing with the challenges associated with being a Muslim in the Netherlands.

The potential to facilitate dialog may also be a particularly powerful asset in the context of the application of the described procedure in the context of P/CVE. As we have noticed at the onset of the article, current P/CVE practice all too often takes an oppositional stance to the communities that are subjected to the P/CVE programs, suggesting there are narratives to be countered and alternative values to be promoted. But such an approach focusses on threat and undermines trust and thereby the chances of success. Affirmation and dialog constitute essential elements for any P/CVE approach to have a truly constructive impact (Ellis and Abdi, 2017). In this sense, the presently described procedure may provide a radical new look on P/CVE practice.

## Data Availability Statement

The raw data supporting the conclusions of this article will be made available by the authors, without undue reservation.

## Ethics Statement

The studies involving human participants were reviewed and approved by the Leiden University Faculty of Governance and Global Affairs. The patients/participants provided their written informed consent to participate in this study.

## Author Contributions

The authors share their contribution in idea development, research design, and research execution. Data analysis and reporting was done by MD and checked by JA. All authors contributed to the article and approved the submitted version.

### Conflict of Interest

The authors declare that the research was conducted in the absence of any commercial or financial relationships that could be construed as a potential conflict of interest.
